# Osteoporosis treatment: current drugs and future developments

**DOI:** 10.3389/fphar.2024.1456796

**Published:** 2024-08-12

**Authors:** Ya-jing Chen, Li-hua Jia, Tao-hong Han, Zhi-hui Zhao, Jian Yang, Jun-ping Xiao, Hong-Jun Yang, Ke Yang

**Affiliations:** ^1^ Beijing Key Laboratory of Traditional Chinese Medicine Basic Research on Prevention and Treatment for Major Diseases, Experimental Research Center, China Academy of Chinese Medical Sciences, Beijing, China; ^2^ Department of Urology, Jinhua Hospital of Traditional Chinese Medicine, Affiliated to Zhejiang University of Traditional Chinese Medicine, Jinhua, China; ^3^ Zhejiang Provincial Key Laboratory of Biometrology and Inspection and Quarantine, College of Life Science, China Jiliang University, Hangzhou, China; ^4^ State Key Laboratory Breeding Base of Dao-di Herbs, National Resource Center for Chinese Materia Medica, China Academy of Chinese Medical Sciences, Beijing, China; ^5^ Dexing Research and Training Center of Chinese Medical Sciences, Dexing, China; ^6^ Jiangxi Prozin Pharmaceutical Co., Ltd., Jiangxi, China

**Keywords:** osteoporosis, pharmacotherapy, bone remodeling, inhibition of bone resorption, promotion of bone formation

## Abstract

Osteoporosis is a common systemic metabolic disease characterized by a decrease in bone density and bone mass, destruction of bone tissue microstructure, and increased bone fragility leading to fracture susceptibility. Pharmacological treatment of osteoporosis is the focus of current research, and anti-osteoporosis drugs usually play a role in inhibiting bone resorption, promoting bone formation, and having a dual role. However, most of the drugs have the disadvantages of single target and high toxic and side effects. There are many types of traditional Chinese medicines (TCM), from a wide range of sources and mostly plants. Herbal plants have unique advantages in regulating the relationship between osteoporosis and the immune system, acupuncture therapy has significant therapeutic effects in combination with medicine for osteoporosis. The target cells and specific molecular mechanisms of TCM in preventing and treating osteoporosis have not been fully elucidated. At present, there is a lack of comprehensive understanding of the pathological mechanism of the disease. Therefore, a better understanding of the pathological signaling pathways and key molecules involved in the pathogenesis of osteoporosis is crucial for the design of therapeutic targets and drug development. In this paper, we review the development and current status of anti-osteoporosis drugs currently in clinical application and under development to provide relevant basis and reference for drug prevention and treatment of osteoporosis, with the aim of promoting pharmacological research and new drug development.

## 1 Introduction

Osteoporosis is a common systemic metabolic disease characterized by a decrease in bone density and bone mass, destruction of bone tissue microstructure, and increased bone fragility leading to fracture susceptibility. Osteoporosis is divided into primary osteoporosis and secondary osteoporosis, and primary osteoporosis includes three types of osteoporosis: type I p ostmenopausal osteoporosis, type II senile osteoporosis and idiopathic adolescent osteoporosis. Its prevalence increases with age, and the World Population Prospects 2019 released by the United Nations points out that the proportion of the global population over 65 years of age is expected to rise to 16% by 2050, and the total number of patients with osteoporosis will reach 221 million (Mukherjee and Chattopadhyay, 2016). With an increasingly aging population, osteoporosis has become an important public health issue facing the world. The total annual cost of osteoporotic fractures is estimated to be $20 billion in the United States and $130 billion in the European Union (Cummings and Melton, 2002). Primary osteoporosis is characterized by brittle bones, an increased risk of fracture and the ensuing chronic pain leading to a reduced quality of life for postmenopausal women and older adults, and a heavy burden on society. In a cross-sectional study of 20,416 Chinese, the prevalence of osteoporosis in adults aged 40 years or older was 5.0% in men and 20.6% in women (Wang et al., 2021). In a US study, the prevalence of osteoporosis was found to be 6%–11% and low bone mass 28%–45%, with the prevalence of both diseases significantly higher in women (10%–17% with osteoporosis; 36%–53% with low bone mass) than in men (3%–5% with osteoporosis; 19%–36% with low bone mass) (Looker et al., 2017). Since the late 1980s, when physicians were able to offer estrogen replacement and calcitonin, as well as calcium and vitamin D supplements to postmenopausal women, the pathogenesis of osteoporosis has been largely unraveled and several effective and safe treatments have been developed to treat osteoporosis and prevent fractures (Lorentzon, 2019).

Prevention of osteoporosis can be achieved by consuming the nutrients needed for bone health, vitamin D and calcium from food, which can be the first choice for the prevention and treatment of osteoporosis (Weaver et al., 2016; Paschalis et al., 2017), while anti-osteoporosis drug therapy is necessary to improve bone mass and reduce the risk of fracture. At present, anti-osteoporosis drugs are mainly divided into osteotropic drugs, drugs that promote bone formation and drugs that inhibit bone resorption. In addition, single Chinese medicine and its compound preparations also play an important role. But a single anti-osteoporosis drug cannot be used throughout the entire disease, and a long procedure of consistent treatment should be adhered to, minimizing the combined use of drugs with the same mechanism of action. Understanding and identifying potential biomarkers and therapeutic targets at different stages of osteoporosis. By identifying the main signaling pathways and molecules that play a key role in the occurrence and development of osteoporosis, potential therapeutic targets are selected to develop new drugs. In this review, we first outline the epidemiology of osteoporosis, including its prevalence and burden in men and women. Next, the pathogenesis of osteoporosis is reviewed, which focuses on the regulation of parathyroid hormone and glucocorticoid, the role and regulation of Wnt/β-catenin, RANKL and other pathological signaling pathways and key regulatory factors in the development of osteoporosis. This article mainly reviews the research progress of currently approved clinical therapeutic drugs for osteoporosis, and updates the new drug strategy for future osteoporosis treatment. In addition, the anti-osteoporosis effect of natural Chinese medicine and the combined treatment of acupuncture were also discussed. It will have an important influence and reference value on the formulation of drug treatment strategies for osteoporosis.

## 2 Pathogenesis of osteoporosis

### 2.1 Calmodulin affects bone metabolism

Osteoporosis is caused by a complex interaction between local and systemic regulators of bone cell function and is regulated by multiple factors such as genes, transcription factors, signaling pathways, hormone levels, and cytokines. Some nutrients in the body can be produced by the body, but some cannot. Calcium as an essential nutrient needs to be provided through exogenous sources, mainly from food and absorbed in the small intestine. More than 99% of the body’s calcium is stored in bone tissue and teeth in the form of hydroxyapatite (Weaver and Peacock, 2019), hydroxyapatite performs a critical structural function by providing rigidity to the skeletal system, and it is free cationic calcium that performs the physiological role of blood calcium, accounting for about 45% of total plasma calcium. Serum calcium concentrations are usually controlled by the actions of parathyroid hormone, vitamin D and calcitonin, which regulate intestinal calcium absorption, renal excretion or reabsorption, and removal or admixture into bone tissue, respectively (Shkembi and Huppertz, 2021).

**FIGURE 1:**
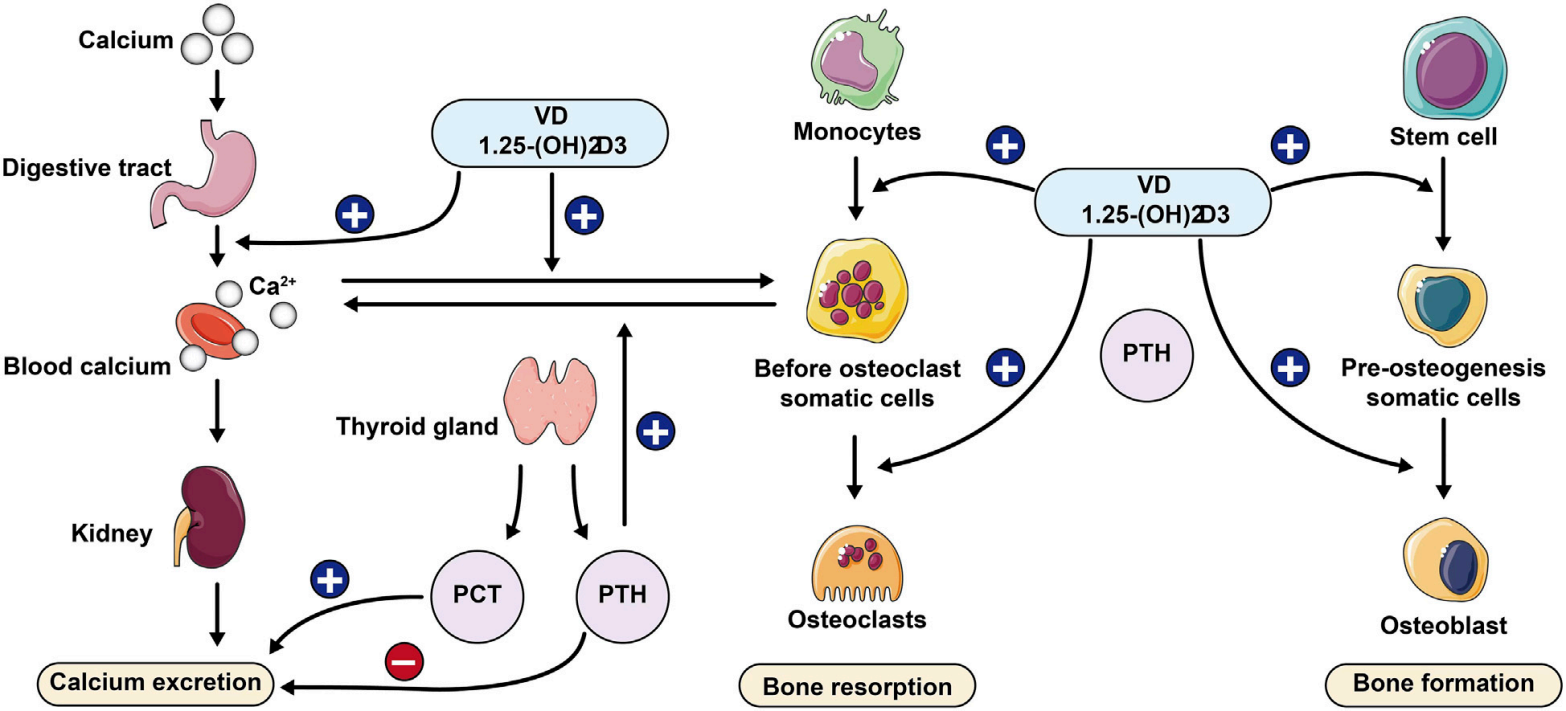
Role of calcium-regulating hormone in bone metabolism PCT: Procalcitonin; PTH: Parathyroid hormone.

Parathyroid hormone (PTH) is a key hormone that regulates several body organs and systems. It is a polypeptide containing 84 amino acids and is mainly expressed by four small glands (parathyroid glands) located behind the thyroid gland, which are dedicated to the control of calcium homeostasis (Corbetta, 2019; Lombardi et al., 2020). PTH stimulation increases the rate of cell proliferation, facilitates the differentiation of MSCs into osteoblasts, reduces osteoblast apoptosis, and promotes bone matrix deposition and mineralization (Lombardi et al., 2020). It also induces RANKL expression in osteoblasts and downregulates osteoclastogenesis inhibitory factor (OPG) expression, thereby stimulating osteoclastogenesis and osteoclast resorption (Boyce, 2013). PTH with calcium and phosphate, promotes bone matrix resorption and the release of other factors involved in matrix synthesis, such as transcription factor (TCF) and bone morphogenetic proteins (BMP), thereby inducing osteoclastogenesis and increasing osteoblast activity (Goltzman, 2018). Thus when dietary supplements are obtained at low levels it can lead to low levels of calcium ions in the body, and increasing age can lead to calcium malabsorption, which in turn can lead to secondary hyperparathyroidism, increasing bone resorption and causing further bone loss. Changes in body calcium and phosphorus levels and bone microenvironmental homeostasis will occur, leading to osteoporosis. The role of calmodulin in bone metabolism is shown in Figure 1.

### 2.2 Bone remodeling maintains homeostasis

Bone cells such as osteoblasts, osteoclasts and osteocytes can play a regulatory role in the body. Promoting bone resorption and bone formation in bone tissue metabolism can form a dynamic balance mechanism. So bone remodeling is essential for maintaining bone mass and systemic mineral homeostasis (Siddiqui and Partridge, 2016). Bone remodeling can be divided into 5 phases: activation phase, osteoclast recruitment and resorption phase, reversal phase, osteoblast recruitment and formation phase, and termination phase (Li et al., 2021), it is regulated by osteoblasts, osteoclasts and osteocytes. Osteoblasts are the cells responsible for bone deposition and mineralization and are derived from mesenchymal stem cells (MSCs). BMPs, growth factors such as fibroblast growth factor (FGF) and insulin like growth factor (IGF), angiogenic factors such as endothelin-1, hormones such as PTH and prostaglandin agonists, all regulate osteoblast differentiation (Qin et al., 2003). Among all pathways regulating MSC proliferation and differentiation, the Wnt/β-catenin pathway is the most important and well-established pathway controlling osteoblast formation and plays an essential role in maintaining bone homeostasis (Pacifici, 2016; Liu et al., 2022). Osteoclasts are responsible for resorption and their differentiation is regulated mainly by monocyte/macrophage colony-stimulating factors, RANK ligands and osteoproteins (M-CSF, RANKL/RANK/OPG) (Teitelbaum, 2000). Osteoblasts can regulate osteoclasts and osteoclasts can affect osteoblast or bone formation activity (Chen et al., 2018). Osteoblasts are important regulators of bone remodeling and can secrete a variety of cytokines, including Dkk1 (antagonist of the Wnt pathway), sclerostin, RANKL, and OPG (21). Overall the whole process of bone remodeling is a tightly controlled and coordinated process regulated by many cell types, and this homeostatic mechanism is disrupted in patients with osteoporosis, leading to their bone loss and susceptibility to fracture. Osteoporosis results when there is insufficient peak bone mass, increased bone resorption and decreased bone formation.

### 2.3 Regulation of hormones

**FIGURE 2:**
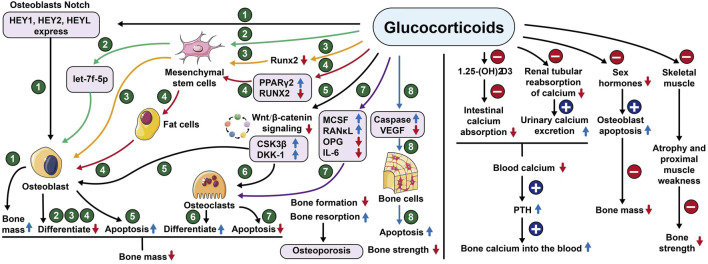
Mechanism of action of glucocorticoids. Notes: HEY1or2 (Hes related family bHLH transcription factor with YRPW motif 1or2), HEYL (Helicophan family gene of transcription factors), let-7f-5p (Members of the pre-miRNA lethal-7), Runx2 (Runt-related transcription factor 2), PPARγ2 (Peroxisome proliferator-activated receptorγ), CSK3β (Glycogen synthase kinase-3), DKK-1 (Dickkopf-1), MCSF (Macrophage colony-stimulating factor), RANκL (Receptor Activator for Nuclear Factor- κB Ligand), OPG (Osteoprotegerin), IL-6 (Interleukin- 6), VEGF (Vascular endothelial growth factor).

Take menopausal women, for example, one of the main causes of primary osteoporosis is the rapid decline of estrogen caused by the stop of menopausal ovarian function (Wu et al., 2021). At menopause, bone loss accelerates rapidly, with an average decrease in bone mineral density of about 10% during the menopausal transition, the rate of decline may be as high as 10%–20% in the 5–6 years before and after menopause, and 25% of postmenopausal women can be classified as having rapid bone loss (Tella and Gallagher, 2014); Secondary osteoporosis is mainly caused by most systemic diseases and organ dysfunction, such as chronic kidney disease, type I diabetes, hyperthyroidism, chronic liver disease, and systemic lupus erythematosus (Sobh et al., 2022). Glucocorticoids are widely used in inflammatory and immune diseases, and their induction is the most common cause of secondary osteoporosis (Coskun Benlidayi, 2018), rapid bone loss occurs after oral glucocorticoid administration, and the risk of fracture is greatly increased. The mechanism of glucocorticoid-induced osteoporosis is complex, with its ability to reduce the conversion of mesenchymal stem cells to osteoblasts and their differentiation into adipocytes (Lane, 2019). The mechanism of glucocorticoid-induced osteoporosis is complex, as it reduces osteoblast formation by upregulating PPARγ2 to activate adipocytes in bone tissue (Adami and Saag, 2019). In addition, glucocorticoids increase the expression of RANKL and macrophage colony-stimulating factor (M-CSF) and decrease the expression of OPG in osteoblasts and osteocytes (El-Gazzar and Högler, 2021). Glucocorticoids inhibit Wnt/β-catenin protein signalling, which has the ability to hinder osteoclast differentiation and promote osteoblast differentiation (Peng et al., 2021). Osteoblasts interact with hematopoietic cells to provide the supportive microenvironment required to maintain erythropoiesis and myelopoiesis (Valderrábano and Wu, 2019; Gaudio et al., 2020). Glucocorticoids can reduce the expression of vascular endothelial growth factor (Liu et al., 2021) and VEGF expression, resulting in reduced skeletal angiogenesis and bone strength. The specific mechanism can be seen in Figure 2.

### 2.4 Regulation of immune system

Recent bone immunology studies have shown that the immune system plays an important regulatory role in the development of osteoporosis and that the skeletal and immune systems share many regulatory molecules, including cytokines, chemokines, receptors and transcription factors (Zhao et al., 2018; Okamoto and Takayanagi, 2019). The immune system can regulate bone metabolism through B-cell, T-cell and many cytokines, as well as osteoblast development and bone turnover through the RANKL/RANK/OPG pathway, but when activated abnormally it can disrupt the balance between osteoblasts and osteoclasts, leading to an imbalance between bone formation and bone resorption (Weitzmann and Ofotokun, 2016; Bozec and Zaiss, 2017; Zhao et al., 2018), which in turn induces osteoporosis.

## 3 Anti-osteoporosis drugs for clinical application

### 3.1 Bone formation promoting drugs

#### 3.1.1 Fluoride

Fluorine is one of the micronutrients necessary to maintain bone growth, it can directly stimulate the proliferation of osteoblasts and inhibit the activity of osteoclasts, thus increasing bone mass (Liu et al., 2019), and it can also induce bone formation by stimulating the differentiation of mesenchymal stem cells towards osteoblasts (Haguenauer et al., 2000). Alkaline phosphatase (ALP) activity is considered a marker of osteoblasts (Rodríguez and Rosselot, 2001), and fluoride regulates bone formation by stimulating ALP activity. Fluoride increases levels of bone cell growth factors such as osteoblast transforming growth factor-β1 (TGF-β1), and ALK5 is a key factor in TGF-β1 signalling. Yang showed that fluoride regulates TGF-β1 signaling in osteoblasts through ALK5 (Yang et al., 2017). Based on these properties, fluoride has been used to treat osteoporosis for nearly 40 years. However, fluoride has a dual effect on bone, inhibiting fluoride-sensitive phosphotyrosine phosphate synthase in osteoblasts at low concentrations and stimulating mitogenic protein kinase mitosis, thereby promoting osteoblast proliferation and increasing osteoblast activity; at high concentrations, it has a toxic effect on osteoblasts and inhibits osteoclast activity (Everett, 2011; Ciosek et al., 2021).

^18^F-NaF is a radiotracer that specifically reflects skeletal blood flow and osteoblast activity in bone tissue or soft tissue (Blake et al., 2018). An emerging imaging modality developed through it in recent years, ^18^F-NaF-PET/CT has the potential to diagnose and monitor skeletal disease early by detecting subtle metabolic changes, which can monitor the molecular effects of osteoporosis to identify age-related osteoporotic changes in the skeleton and be used to better guide treatment decisions (Park et al., 2021).

At present, the common clinical fluoride drugs are sodium fluoride and disodium fluoride phosphate, which should be combined with slow-release preparations in the clinical application process to ensure the safety of drug application. The difference between the tolerated and toxic dose of fluoride in human body is very small (normal range of blood fluoride was 0.15–1.0 mg/L), so the drug dosage should also be strictly controlled.

#### 3.1.2 Parathyroid hormone analogues

Parathyroid hormone is extracted from thyroid secretions and belongs to a group of single-chain polypeptide hormones, which can regulate plasma calcium levels, calcium homeostasis and phosphorus metabolism of the body (Khosla, 2020).

The bone anabolic drug teriparatide, the first FDA-approved bone antimetabolic therapy, is a recombinant human parathyroid hormone amino-terminal 1–34 active fragment, which is biologically active in the same way as human parathyroid hormone, and increases lumbar vertebrae and femoral neck bone mineral density over the long term, and is an effective drug for reducing the risk of vertebral fracture in postmenopausal women with osteoporosis. Previous studies have shown that the reference standard for biochemical markers of bone formation is procollagen type I N-terminal pre-peptide (PINP), and for bone resorption is the C-terminal cross-linking telopeptide (CTX) of serum type I collagen (Vasikaran et al., 2011). Clinical trial shows sustained increase in PINP 3 months after starting teriparatide (Krege et al., 2014). Tsai (Tsai et al., 2019) study showed that the combination of denosumab and high-dose teriparatide resulted in substantial increases in aBMD and vBMD of the hip and spine, increased bone strength, and reduced the risk of fragility fractures. In addition, the combination of teriparatide with other bone resorption inhibitors such as denosumab significantly increased bone mineral density (BMD), and the combination with bisphosphonates such as alendronate diminished the inhibitory effect of bisphosphonates on bone turnover levels (Figliomeni et al., 2018). However, teriparatide is currently sold at a higher price. Abaloparatide, a novel analogue of parathyroid hormone-related protein, became the second osteoantimetabolic therapy for osteoporosis in 2017. Phase III clinical trials reported that in osteoporotic postmenopausal women with low serum CTX levels (a marker of bone resorption), abaloparatide (80 μg/day) and teriparatide (20 μg/day) similar in increasing bone mineral density (Miller et al., 2016). Le Henaff et al. (2020) showed that the same dose of abaloparatide was as effective as PTH (Teitelbaum, 2000; Cummings and Melton, 2002; Qin et al., 2003; Boyce, 2013; Tella and Gallagher, 2014; Chen et al., 2015; Mukherjee and Chattopadhyay, 2016; Pacifici, 2016; Siddiqui and Partridge, 2016; Weaver et al., 2016; Looker et al., 2017; Paschalis et al., 2017; Chen et al., 2018; Coskun Benlidayi, 2018; Goltzman, 2018; Zhao et al., 2018; Adami and Saag, 2019; Corbetta, 2019; Lane, 2019; Lorentzon, 2019; Okamoto and Takayanagi, 2019; Valderrábano and Wu, 2019; Weaver and Peacock, 2019; Gaudio et al., 2020; Lombardi et al., 2020; El-Gazzar and Högler, 2021; Li et al., 2021; Liu et al., 2021; Peng et al., 2021; Shkembi and Huppertz, 2021; Wang et al., 2021; Wu et al., 2021; Liu et al., 2022; Sobh et al., 2022) as a bone antimetabolite by mouse experiments.

#### 3.1.3 Statins

Statins inhibit endogenous cholesterol production and are commonly used as clinical lipid-lowering agents. The use of low-dose statins increases bone resorption, while high-dose statins increase bone formation (Maritz et al., 2001). The main representative drugs lovastatin and simvastatin can reduce the production of mevalonate. Simvastatin exhibits anabolic effects on bone by inhibiting osteoblast apoptosis and inhibiting the differentiation and activity of osteoclasts (Guo et al., 2020). Cheon et al. (2021) showed that pitavastatin can inhibit RANKL-induced osteoclast formation and function by inhibiting Akt, NF-κB and MAPK signaling pathways, leading to the downregulation of c-Fos and NFATc1, major transcription factors in osteoclastogenesis. In addition, statins act by inhibiting the endogenous synthesis of cholesterol, which is the main substrate for sex hormone synthesis, and Leutner et al. (2019) showed a link between sex hormone levels and statins in the pathogenesis of osteoporosis. It is unclear whether HMG-CoA-reductase inhibition plays a role in the pathogenesis of osteoporosis, but the use of statins has been shown to be effective in patients with osteoporosis combined with cardiovascular and cerebrovascular disease.

#### 3.1.4 Androgens and prostaglandins E2

Androgens have the function of promoting bone cell proliferation, accelerating bone protein synthesis and bone mineralization, and increasing the volume of bone trabeculae and bone mass. Decreased androgen levels in the body will cause increased bone resorption and decreased bone formation, leaving bone metabolism in a negative equilibrium, leading to decreased bone density and inducing osteoporosis. Bone density in men is positively correlated with their blood testosterone levels, and serum testosterone levels decline with age (Adler, 2014). Testosterone deficiency predisposes to osteoporosis in men. The American College of Endocrinology 2012 edition guidelines recommend that testosterone replacement therapy should be combined with medications that have anti-osteoporotic fracture efficacy, especially in patients at high risk of fracture (Watts et al., 2012). However, testosterone replacement therapy has many adverse effects, and currently selective androgen receptor modulators (SARMs) show skeletal and muscle anabolic activity in preclinical models (Vajda et al., 2009). The enhancement of muscle mass and strength by SARMs may reduce the risk of fracture due to falls, and oral bioavailable SARMs are a unique alternative for the treatment of osteoporosis (Gao and Dalton, 2007). This class of drugs has shown good efficacy in men with osteoporosis caused by glucocorticoid administration.

Prostaglandin E2, a class of unsaturated fatty acids with a wide range of physiological activities, is a potent osteoanabolic agent that promotes bone synthesis and increases bone mass by stimulating osteoblast differentiation and proliferation. Bone is rich in sensory nerves and interacts with the central nervous system. Chen et al. (2019) showed that prostaglandin E2 (PGE2) secreted by osteoblasts can activate PGE2 receptor four in sensory nerves and regulate bone formation by inhibiting sympathetic nerve activity through the central nervous system.

#### 3.1.5 Strontium salt

Strontium is a trace element found in human bones. Strontium salt can maintain the rate of bone renewal, promote the proliferation and differentiation of osteoblasts, inhibit the formation and differentiation of osteoclasts and promote their apoptosis while maintaining bone formation, leading to reduced bone resorption, and can improve the mechanical strength of bones without changing the structure of bone (Chu et al., 2017), it is a drug with bidirectional regulation of bone metabolism. Strontium can also regulate MSCs, increase the rate of bone formation and the synthesis of collagen and non-collagen proteins in the bone matrix to exert its anti-osteoporosis effect (Kołodziejska et al., 2021). Studies have shown that strontium salts are effective in treating postmenopausal osteoporosis induced by glucocorticoid drugs. Strontium ranelate, a representative drug, reduces the risk of osteoporosis-related fractures by simultaneously inhibiting bone resorption and promoting bone formation. Reginster et al., (2005) study indicated that strontium ranelate-treated patients had a 16% reduction in the relative risk of all non-vertebral fractures and a 19% reduction in the relative risk of major fragility fractures, compared with the placebo group.

In addition, strontium acts through calcium-sensitive receptor (CaSR) receptors in bone tissue cells. In recent years, there have been studies on the introduction of strontium ions in ceramics instead of calcium ions for use as a bone replacement material in the treatment of fractures and defects caused by osteoporosis (Kołodziejska et al., 2021). Mesoporous bioactive glass (MBG) scaffolds obtained by amine doping in strontium can reduce reactive oxygen species levels in bone marrow mesenchymal stem cells in an osteoporosis model by activating the cAMP/PKA signalling pathway, which promotes osteogenesis and acts as an anti-osteoporotic agent (Wu et al., 2022). However, strontium use increases the risk of myocardial infarction, thromboembolic events, and rare severe cutaneous reactions (DRESS syndrome), and therefore has not been approved in the United States (Khosla and Hofbauer, 2017).

### 3.2 Bone resorption inhibiting drugs

#### 3.2.1 Calcitonin

Calcitonin, a peptide hormone secreted by thyroid C cells, is a potent inhibitor of bone resorption. Its family peptide, tryptic amyloid peptide, is able to inhibit osteoclast activity (Naot et al., 2019). Calcitonin receptor (CTR) is a class B G protein-coupled receptor (GPCR) that regulates calcium homeostasis and bone turnover upon activation by calcitonin and is an osteoporosis drug target (Lee et al., 2020). Although the physiological role of calcitonin is to inhibit osteoblast bone formation, the calcitonin receptor is expressed only in osteoclasts and its effect on osteoblasts may be indirect and mediated through osteoclasts. Wnt10b has been shown to be a potential novel therapeutic target for postmenopausal osteoporosis and chronic kidney disease (CKD) related skeletal diseases. Hsiao et al. (2020) showed through mouse experiments that calcitonin induced bone formation by increasing Wnt10b expression in osteoclasts in ovariectomy-induced osteoporosis rats. In addition, calcitonin also acts on specific receptors in the central sensory area and inhibits the release of central pain mediators.

Salmon calcitonin (MI), a peptide hormone that promotes bone remodelling and inhibits bone resorption, has a higher affinity than human calcitonin and is currently used as a clinically indicated osteoporosis drug (Lee, 2021). Tsukamoto et al. (2016) demonstrated through *in vivo* animal experiments that egakunin (EL) causes bone marrow cells to highly express the bone characterising calcitonin receptor and inhibits the differentiation of pro-osteoclasts into osteoclasts. However, calcitonin has limited efficacy in preventing fractures, and long-term use of calcitonin may have an increased risk of cancer.

#### 3.2.2 Estrogenic

Osteoporosis is closely related to estrogen levels, which regulate skeletal homeostasis through direct effects on the immune system and oxidative stress, as well as on bone cells, and can increase bone mass, effectively regulate the excessive bone turnover rate and improve bone density (Weitzmann and Pacifici, 2006). The binding of selective estrogen receptor modulators (SERMs) to estrogen receptors leads to different changes in the receptor spatial conformation (Segura-Uribe et al., 2017), reduce bone loss and increase bone density by activating and/or blocking estrogenic pathways.

Raloxifene is the first selective estrogen receptor modulator approved by the FDA for postmenopausal osteoporosis, primarily for postmenopausal women with significantly decreased levels of estrogen production. Raloxifene inhibits osteoclast activity through multiple signaling pathways, but does not increase the risk of endometrial cancer (Uemura et al., 2019). Raloxifene is effective in reducing the risk of vertebral fracture in patients with osteoporosis (Ayub et al., 2021). The overall safety of raloxifene is good, but it is a hydrophobic molecule and has low water solubility, so it is mainly administered orally as a tablet, resulting in low bioavailability and requiring long-term, high-dose administration to be effective (Khorsand et al., 2018). Yang et al. (2022) encapsulated raloxifene in human serum albumin (HSA)-based nanoparticles as an intravenous drug formulation, which significantly increased bioavailability and improved its half-life in plasma, and could be a potential nanomedicine for the treatment of postmenopausal osteoporosis. Currently, most clinical treatments for postmenopausal osteoporosis are based on estrogen replacement therapy, but long-term use of estrogen increases the risk of breast cancer, cervical cancer and uterine corpus cancer in women.

#### 3.2.3 Bisphosphonates

Bisphosphonates (BPs) are stable pyrophosphonate analogs characterized by the presence of P-C-P groups and are currently used as first-line drugs for the prevention and treatment of osteoporosis in postmenopausal women and men to reduce bone pain and fractures. BPs promote osteoprotegerin secretion by osteoblasts, and their mechanism of action to inhibit bone resorption is mainly twofold. On the one hand, they regulate osteoclast proliferation, differentiation and apoptosis, and on the other hand, they inhibit bone resorption by inhibiting osteoclast-mediated expression of cytokines such as interleukin-6 or tumor necrosis factor (Russell et al., 2008). The representative drugs include alendronate, zoledronate and risedronate.

Alendronate increases BMD and reduces fracture risk in osteoporotic patients. A Meta-analysis showed that Barrionuevo et al. (2019), in older women with a higher risk of fracture, alendronate reduced the risk of vertebral fracture by 44%, hip fracture by 40%, and non-vertebral fracture by 17% compared with the placebo group. Zoledronate is used to treat osteolysis caused by malignant tumor bone metastases with good affinity for bone and long duration of action. Hortobagyi et al. (2019) found in three independent studies that zoledronate in combination with other antitumor therapies for the treatment of bone metastases from malignant tumors may not cause bone saturation. Boonen et al. (2012) demonstrated that zoledronate reduced the incidence of vertebral fractures by 67%. Risedronate is effective in increasing BMD in men with primary and corticosteroid-induced osteoporosis and is able to prevent vertebral fractures within the first year of treatment in corticosteroid-treated men (Bobba and Adachi, 2007). Mawatari et al. (2017) study confirmed that risedronate was effective in increasing BMD and reducing fracture risk in the lumbar spine. Overall, bisphosphonates are extremely effective drugs for the treatment of osteoporosis, but they may have side effects of atypical femur fractures and osteonecrosis of the jaw. In patients with severe osteoporosis and high fracture risk, it is difficult to use bisphosphonates alone for long-term fracture prevention and recovery of BMD. patients may be better served by starting with a bone-building drug such as teriparatide followed by an anti-resorptive drug for long-term fracture prevention (Lorentzon, 2019). Characteristics of various generations of bisphosphonates are shown in Table 1.

**TABLE 1:** Characteristics of various generations of bisphosphonates.

Drugs name	Algebra	Features	Fracture impact points	Medication	Adaptation Type	Bibliography
Etidronate disodium	First Generation	Nitrogen-free simple structures Multiple adverse effects and inhibition of bone mineralization in patients		Oral 400 mg/day	Postmenopausal osteoporosis, age-related osteoporosis	Heaney and Saville (1976)
Disodium clodronate				Intravenous drip, oral 300 mg/day	Multiple types of osteoporosis	Moretti et al. (2021)
Pamidronate disodium	Second Generation	Contains amino acids Increased anti-bone resorption effect		Intravenous drip 30–60 mg/each time	Malignant tumor complicated by hypercalcemia, osteolytic cancer metastasis with bone pain type osteoporosis	Xu et al. (2017)
Alendronate sodium			Vertebral body, non-vertebral body, hip joint	Oral 10 mg/day	Postmenopausal osteoporosis, male osteoporosis, glucocorticoid-induced osteoporosis	Cummings et al. (2020)
Zoledronic sodium	Third Generation	Heterocyclic or containing saturated hydrocarbon chains Strong anti-bone resorption effect, safe for clinical application	Vertebral body, non-vertebral body, hip joint	Intravenous injection 5 mg/year	Male osteoporosis, glucocorticoid-induced osteoporosis, bone metastasis from malignant tumors	Harding et al. (2010)
Risedronate sodium			Vertebral body, non-vertebral body, hip joint	Oral 5 mg/day	Postmenopausal osteoporosis, glucocorticosteroid osteoporosis	McClung and Ebetino (2020)
Ibandronate sodium			Spine	Oral, intravenous injection 2.5 mg/day	Postmenopausal osteoporosis	Keating (2016)

### 3.3 Bone nutrient drugs

#### 3.3.1 Calcium with vitamin D and its derivatives

Calcium is the main element in the composition of human bones, and free calcium ions are essential for maintaining bone mineralization and participating in bone metabolism. Osteoporosis is mainly caused by calcium deficiency, especially in the elderly due to aging or disease leading to intestinal calcium absorption obstacles, calcium deficiency can be increased by supplementing calcium carbonate, calcium citrate, calcium gluconate and other calcium supplements to increase the intake of calcium. Al-Dujaili et al. (2016) found in animal experiments that the levels of osteoblast-specific markers were significantly decreased in mice with specific knockout of the calcium ion receptor gene compared to wild-type mice, accompanied by a decrease in bone mass, suggesting that calcium ions have a role in promoting osteoblast differentiation and proliferation.

Vitamin D promotes the body’s absorption and storage of calcium ions and has a direct impact on the balance of calcium and phosphorus in the body. Vitamin D metabolizes osteotriol, a calcium-regulating hormone circulating in the body, which accelerates gene synthesis in osteoblasts and promotes bone mineralization. Secondary hyperparathyroidism manifests as a relative deficiency of vitamin D. Active vitamin D is able to act directly on the parathyroid glands and reduce parathyroid hormone synthesis and secretion, and also binds to vitamin D receptors in osteoblasts and osteoclasts, ultimately promoting skeletal calcium and phosphorus deposition and mineralization. A Meta-analysis (DIPART Vitamin D Individual Patient Analysis of Randomized Trials Group, 2010) demonstrated that vitamin D alone is not effective in preventing fractures, however, vitamin D in combination with calcium reduces the incidence of fractures to a certain extent.

#### 3.3.2 Other vitamin classes

Vitamin C is an important substance for bone metabolism. When the body is deficient in vitamin C, the metabolism of protein and polysaccharides in bones will be impaired to varying degrees, affecting the development and growth of bones, resulting in osteoporosis. Vitamin C can also be combined with calcium ions in the intestinal tract, and appropriate levels of vitamin C in the diet are beneficial to bone metabolism and the absorption of calcium ions (Brzezińska et al., 2020). In addition, vitamin C intake increases the expression of osteoblast differentiation genes, thus stimulating bone formation (Zhu et al., 2012). Choi et al. (2019) found that vitamin C inhibits osteoclastogenesis and promotes osteoblast formation through the Wnt/β-Catenin/ATF4 signaling pathway, thereby inhibiting osteoporosis.

Vitamin K analogs are able to act in multiple bone metabolic pathways and maintain normal levels of carboxylated (active form) vitamin K-dependent proteins (osteocalcin and matrix Gla protein) by stimulating osteoblast differentiation, upregulating bone marker gene expression and thereby promoting bone formation and inhibiting bone resorption (Fusaro et al., 2020). Vitamin K2 administration improves lumbar spine bone density and substantially reduces vertebral fracture rates in postmenopausal women. Iwamoto (2014) showed by reviewing randomized controlled trials in the literature that treatment with a synthetic vitamin K2 drug called menaquinone reduced serum carboxylated osteocalcin (ucOC) concentrations, modestly increased lumbar spine BMD, and reduced the incidence of vertebral fractures. Currently, menaquinone is a second-line drug for the treatment of postmenopausal osteoporosis, and its efficacy in combination with bisphosphonates for the treatment of fractures in women with postmenopausal osteoporosis needs to be further explored. Clinically used anti-osteoporosis drugs are shown in Table 2.

**TABLE 2:** Clinically used anti-osteoporosis drugs.

Classification	Drug type	Representative drugs	Scope of adaptation	Adverse reactions	Bibliography
Bone formation promoting drugs	Fluoride	Disodium fluorophosphate	Postmenopausal osteoporosis; some secondary osteoporosis	High concentrations are likely to cause bone cancer and neuroarthritis	Haguenauer et al. (2000)
	Parathyroid hormone-like compounds	Teriparatide	Postmenopausal osteoporosis; reduced risk of vertebral and non-vertebral fractures	Orthostatic hypotension, tingling sensation in the extremities	Blick et al. (2008)
	Statins	Simvastatin	Osteoporosis in combination with cardiovascular disease	Liver injury	Wu et al. (2020)
	Androgen	Testosterone	Glucocorticoid-induced osteoporosis in men	Cardiovascular and cerebrovascular injuries	Vescini et al. (2021)
	Strontium salt	Strontium ranelate	Postmenopausal osteoporosis; glucocorticoid osteoporosis; reduced risk of vertebral, non-vertebral, fragility fractures	Myocardial infarction, thromboembolism, DRESS syndrome	O Donnell et al. (2006)
Bone resorption inhibiting drugs	Calcitonin	Salmon calcitonin	Men and postmenopausal osteoporosis; reduced risk of vertebral fracture	Allergic reactions, hypocalcemia	Henriksen et al. (2010)
	Estrogenic	Raloxifene	Postmenopausal osteoporosis; reduced risk of vertebral fracture	Thrombocytopenia, gastrointestinal reactions	D Amelio and Isaia (2013)
	Bisphosphonates	Alendronate sodium		Skeletal muscle pain	Cummings et al. (2020)
Bone nutrition drugs	Calcium	Calcium carbonate	Prevention and treatment of osteoporosis	Constipation, hypercalcemia	
	Vitamin D and its derivatives	Vitamin D3	Preventing and treating osteoporosis; combining calcium supplements to reduce fracture risk		Avenell et al. (2014)
	Vitamin K	Osteotriol	Postmenopausal osteoporosis; reduced risk of vertebral fracture	Hepatotoxicity, hemolytic anemia	Lanham-New (2008)

### 3.4 New anti-osteoporosis drugs

The previous approach to the development of new therapies for osteoporosis drugs was from the discovery of animal studies and clinical observations (estrogens, calcitonin and teriparatide) or the repurposing of existing compounds (bisphosphonates), which shifted after 2000 with the discovery of denosumab to a subcellular assessment through careful analysis of rare skeletal diseases and bone cell biology, especially osteoclasts, which emphasizes the translational research importance of transformation research (Khosla and Hofbauer, 2017).

#### 3.4.1 Denosumab

Denosumab is a humanized IgG2-type monoclonal antibody analog that acts against RANKL protein and is recommended as an alternative to bisphosphonates in the treatment of postmenopausal osteoporosis. It is a RANKL inhibitors (Zhang et al., 2020), since RANK ligand is a transmembrane or soluble protein necessary for osteoclasts to maintain their structure, function and survival, Denosumab inhibits RANKL-RANK-OPG signaling pathway-mediated osteoclast differentiation and activation by competitively binding to RANKL protein and exerting an osteoprotective effect similar to that of osteoprotectin. Denosumab inhibits osteoclast differentiation and activation mediated by the RANKL-RANK-OPG signaling pathway, substantially reducing the number of osteoclasts, thereby reducing bone resorption and improving bone mass and quality (Kostenuik et al., 2009). Denosumab was also highly effective in enhancing BMD values and reducing bone turnover indexes, and clearly reduced fracture incidence at multiple target sites (Bone et al., 2017). The phase III randomized FREEDOM trial found that Denosumab maintained fracture rates at low levels, and using quantitative CT, Denosumab significantly increased the bone mineral content of cortical, cancellous, and subcortical bone in the lumbar spine, hip, and radius, and increased the strength of the femur and lumbar spine. Denosumab was approved in 2010, but only one RANKL antibody, TK006, is currently in phase I clinical trials and some side effects have been observed in the treatment of bone metastases from breast cancer.

#### 3.4.2 Romosozumab and blosozumab

Anti-osteoporosis drug targets are currently emerging, but most drugs in clinical trials are still focused on conventional targets. Sclerostin, a 22-kDa glycoprotein secreted primarily by osteoblasts, is a soluble inhibitor of regulated Wnt signaling (Maré et al., 2020). Therefore, when present at increased concentrations, it leads to increased bone resorption and decreased bone formation. Treatment with the sclerostin antibody romosozumab increases BMD more significantly and rapidly than alendronate and is also superior to alendronate in reducing the risk of vertebral and nonvertebral fractures in postmenopausal women with osteoporosis. Blosozumab is a synthetic IgG-type monoclonal sclerostin antibody, structurally different from romosozumab, that has been shown to be effective in phase II phase II trial (Li et al., 2021). Preclinical studies have not detected any soft tissue mineralization as a result of anti-sclerostin antibody treatment, but the role of sclerostin in ectopic calcification has not been fully elucidated. Romosozumab may increase the risk of myocardial infarction, and cardiovascular death, and should be used with caution in osteoporotic patients with cardiovascular disease.

#### 3.4.3 Odanacatib

M-CSF and RANCL are two key factors in osteoclast differentiation and activation that catalyze the deconstruction of osteoclast biology, and new targets have been identified through studies of transcription factors and key components of osteoclast structure and function, among which histone lyase K has received widespread attention. Histone K is secreted by osteoblasts, is highly expressed in osteoclasts, and its inhibition reduces bone resorption, thus making this enzyme an attractive target for antiresorptive osteoporosis therapy (Lu et al., 2018). Odanacatib is a selective, reversible inhibitor of tissue proteinase K. The drug consistently increases lumbar spine and hip BMD and reduces fracture risk. It also reduces serum type I collagen carboxy-terminal peptide and urinary type I collagen amino-terminal peptide levels, but does not significantly reduce bone formation marker levels (Stone et al., 2019). Papapoulos et al. (2021) prospectively assessed the location and incidence of all femoral fractures in women at increased risk of fracture during treatment with the tissue proteinase K inhibitor ODN or placebo in a long-term ODN fracture trial using predefined criteria and found that the incidence of femoral fractures and hip fractures was lower in the ODN group than in the placebo group.

#### 3.4.4 TGF-β/BMP superfamily members

Bone tissue maintains its metabolic stability by self-renewal through bone turnover mechanisms. During bone turnover, many systemic endocrine factors and local cytokines are involved in the regulation of this process: BMPs are abundantly present in the bone matrix and have been found to cause ectopic osteogenesis in muscle (Lowery and Rosen, 2018; Gomez-Puerto et al., 2019). Platelet-derived growth factors (PDGFs) are a class of growth factors that promote cell proliferation and migration and have important roles in the control of embryonic development and adult tissue homeostasis, as well as in bone development, and the absence of PDGFRα leads to defects in skeletal and reproductive development, which can act on bone marrow mesenchymal stem cells and allow the cells to play an important role in fracture repair (Peng et al., 2020). IGF, mainly IGF-Ⅰ and IGF-Ⅱ, is a broad-spectrum growth-promoting factor. Physiological concentrations of IGF enhance collagen fiber production and bone matrix biosynthesis, and promote osteoblast proliferation and differentiation (Tzanakakis et al., 2021). Physiological concentrations of IGF can enhance collagen fiber production and bone matrix biosynthesis, and promote osteoblast proliferation and differentiation.

BMP belong to the same superfamily as transforming growth factor beta (TGFβ), growth and differentiation factor (GDF) and activin-related superfamilies. They are inducers of bone formation and are now known to be involved in morphogenetic activities and cellular differentiation throughout the body, including adipose tissue development and adipose differentiation (Blázquez-Medela et al., 2019), previous studies have demonstrated the relatively definitive efficacy of synthetic human bone BMP-2 and TGF-β etc. In osteoinduction of bone regeneration and promotion of fracture healing. With the emergence of new findings in cell signaling, the TGF-β/BMP superfamily member from liver products BMP-9 has received new attention due to its unique potential to induce osteogenic differentiation of stem cells, and it was found that BMP-9 may be one of the most potent molecules in the bone morphogenetic protein family to induce osteogenic differentiation of stem cells. The Smad cell signaling pathway triggered by BMP is normally inhibited by Noggin. However, BMP-9 is resistant to Noggin and, therefore, contributes to a more robust differentiation of osteoblasts from bone progenitor cells. Recent advances in non-viral BMP-9 delivery also highlight the efficacy of the protein molecule at low doses as a more effective and safer alternative to BMP-2 (Bharadwaz and Jayasuriya, 2021). Another TGF-β superfamily member, activin A, is an important positive RANKL-induced osteoblastogenesis receptor activator, in which Spad-mediated signaling is essential for the induction of osteoclast development and function, activin A may be a good candidate for the treatment of CKD-induced osteomalacia or osteoporosis and is expected to be a new therapeutic target for osteoporosis (Sugatani, 2018).

## 4 Theory of osteoporosis treatment in Chinese medicine

According to its clinical symptoms, this disease belongs to the category of “paralysis”, “bone paralysis”, “bone impotence” and “bone dryness”. Among them, “bone impotence” is the closest to osteoporosis, so “bone impotence” is generally used as the diagnostic name for osteoporosis. Chinese medicine believes that the kidney is the origin of the first day and the spleen is the origin of the second day, and the two are interdependent and affect each other. The kidney collects essence and produces marrow, the spleen is responsible for growth and development, and the bone marrow depends on nourishment; kidney deficiency, spleen deficiency, and liver qi depression can all lead to atrophy of the bones, i.e., osteoporosis (Zhao and Wang, 2003). Kidney deficiency affects calcium and phosphorus metabolism and thus decreases bone density, and affects bone metabolism in several ways. On the one hand, kidney deficiency causes endocrine dysfunction, “kidney deficiency-hypothalamus-pituitary-adrenal (HPA) axis dysfunction”; On the other hand, kidney deficiency causes changes in trace elements in the body, lowering serum zinc levels and affecting bone growth and development and systemic tissue function. Therefore, according to TCM kidney theory many herbs that nourish the kidneys have been found to restore bones and can be used to treat bone related disorders.

Chinese medicine is different from modern medicine in practice and theory. Chinese medicine treatment is mainly to enhance the kidney’s main bone and marrow production, the liver’s main tendon, the spleen’s main transportation and the nourishing effect of essence, qi, blood, fluid and fluid on bone, which can regulate the body’s hormone level, strengthen the activity of osteoblasts, keep the bone volume stable, and make the bone mineral density gradually regain. In general, the main treatment of TCM is to nourish the kidney and fill the essence, supplemented by strengthening the liver and spleen, activating blood circulation and relieving pain (Yuan et al., 2022). Chinese medicine mainly inhibits osteoclast activity and bone resorption to combat osteoporosis. Current TCM treatment focuses on kidney deficiency, spleen deficiency and blood deficiency, and the clinical use of medicine is basically based on the treatment principles of tonifying the kidney and strengthening the bones, tonifying the spleen and kidney, tonifying the liver and kidney, and activating blood circulation and removing blood stasis. A search of the literature on herbal medicines in osteoporosis and bone regeneration revealed that herbal control can significantly improve bone density in osteoporosis patients (Li et al., 2022). Herbal medicines are widely used in China for the treatment of various types of orthopaedic diseases, especially osteoporosis and fractures. However, their benefits and harms have not been fully and definitively assessed. In addition, TCM, as a discipline of traditional healing, includes acupuncture and moxibustion, medicinal food and massage. Among them, acupuncture has attracted attention for producing effective effects on osteoporosis (Peng et al., 2022).

### 4.1 Natural Chinese medicine products

As a possible alternative to anti-osteoporosis drugs, natural herbal products have received increasing attention from scientific research. Chinese herbal medicines are products made from any part of medicinal plants (leaves, stems, buds, flowers or roots). Sometimes non‐plant based components (for example, insects, deer horn, snake, various shells and powdered fossil) are included. These can be used in the form of raw plant materials, or water or alcohol extracts of raw plant materials (Liu et al., 2014). They contain a variety of active ingredients such as total flavonoids, polysaccharides, saponins and alkaloids, which mainly play a role in the treatment of osteoporosis by regulating the body’s hormone levels and calcium and phosphorus metabolism, anti-inflammatory response, inhibiting osteoclast activity, enhancing osteoblast proliferation and improving bone microstructure (Wang et al., 2013; He et al., 2019; Peng et al., 2022).

#### 4.1.1 Anti-osteoporotic active ingredients of TCM

Total flavonoids play an important role in the treatment of osteoporosis as the main active ingredient of many herbs. The Chinese herb Epimedium is commonly used for postmenopausal osteoporosis and is mainly the dried leaves of Epimedium, family Berberaceae. *In vivo* and *in vitro* experiments have demonstrated that the various active chemical components of Epimedium, including flavonoids, lignans, phenol glycosides, alkaloids, polysaccharides, and volatile oils, have antioxidant, antitumor, anti-aging, anti-inflammatory, antiviral, antibacterial, anti-atherosclerotic, antidepressant, and anti-hepatotoxic effects (Shi et al., 2022). In the treatment of osteoporosis, it has the effect of tonifying kidney yang, strengthening tendons and bones, as recorded in the Compendium of Materia Medica: “Epimedium, which is warm in nature but not cold, can benefit essence, and is suitable for those who are deficient in true yang”. As the main active ingredient of Epimedium, total flavonoids can improve the differentiation of bone marrow mesenchymal stem cells to osteoblasts, stimulate osteoblasts to produce more bone matrix, and play a role in promoting bone formation (Zhang D. et al., 2016; Yang et al., 2019). Bone tonic is an important active ingredient that promotes osteogenic differentiation of bone marrow mesenchymal stem cells and inhibits osteoclast-mediated bone resorption. Bone tonic also has a beneficial effect on fracture healing by promoting calcium absorption in bone and increasing blood calcium and phosphorus levels. Bone tonic can inhibit bone resorption or promote bone formation by regulating bone metabolism-related molecular signaling pathways, such as the OPG/RANKL/RANK pathway, histone K pathway, Wnt/β-catenin pathway, and bone formation protein pathway (Jin et al., 2022). Psoralea isoflavones have an inhibitory effect on osteoclast differentiation and function, inhibiting RANKL-mediated activation of the NF-κB pathway, which in turn affects osteoclast differentiation (McClung et al., 2019). It was found to reduce osteoporosis symptoms in mice after ovariectomy.

Different natural products contain different anti-osteoporotic active substances. For example, *Dipsaci Radix* saponin can induce differentiation of rat bone marrow mesenchymal stem cells toward osteoblasts and promote osteoblast differentiation. The results of the mouse experiments showed that the consumption of Radix Dipsaci extract caused a 4.50% increase in bone volume/tissue volume ratio. The bone trabeculae increased by 11.82% in number so that the bone density was increased (Wong et al., 2007). Cornus officinalis and its active constituents modulate the function, activity, and quantity of OC by inhibiting MAPK, AKT, ERK, JNK signaling pathways to reduce the expression of OC-related proteins RANKL, c-Fos, NFATc1 and CTSK, thus inhibit bone resorption. These novel findings designate Cornus officinalis and its active constituents as potential therapeutic anti-OC targets for the treatment of OP (Tang et al., 2023). Icariin significantly inhibited the expression of RANK m RNA, which led to the conclusion that Icariin inhibited the differentiation of osteoclasts by suppressing the production of osteoclast β-actin, and then downregulated the expression of RANK on the surface of osteoclasts and their precursor cells, acting on the RANK-mediated nuclear factor k B signaling pathway (Wang et al., 2018).

#### 4.1.2 Phytoestrogen-like effects

Phytoestrogens of natural origin are the current focus of new drug development. Phytoestrogens are a class of non-steroidal compounds isolated from plants that can bind to the body’s estrogen receptors and produce estrogen-like effects, and their chemical structures are similar to 17β-estradiol (Rietjens et al., 2017). Phytoestrogens have a variety of physiological activities and can bind to estrogen receptors *in vivo* and exert bidirectional regulatory effects. Phytoestrogens can prevent and treat postmenopausal osteoporosis through various mechanisms of action, promote bone formation and inhibit bone resorption, and have important effects on bone metabolism in bone marrow mesenchymal stem cells (BMSCs) (Jayusman et al., 2023). Both experimental and clinical studies have shown that Chinese herbal medicines such as Angelica sinensis, Eucommia globulus and Astragalus Membranaceus, which are currently attracting widespread attention in the prevention and treatment of osteoporosis.

Eucommia globulus acts on the endocrine system, circulatory system, nervous system and immune system through multi-component, multi-target and multi-pathway to regulate bone metabolism. In dexamethasone (Dex)-induced osteoporosis mice, EuOCP3 treatment restored cortical bone thickness, increased mineralized bone area, enhanced the number of osteoblasts, and decreased the number of osteoclasts on the surface of cortical bone (Song et al., 2023). The effect of Angelica sinensis on osteoporosis may be related to its antioxidant effect and estrogen-like effect. An 8-week administration of angelica effectively upregulated serum estradiol levels, reduced the ratio of bone alkaline phosphatase (BALP) to total bone alkaline phosphatase (TALP), and promoted bone formation. Molecular structure of mullein isoflavone extracted from Astragalus membranaceus is similar to estradiol and may exhibit estrogen-like effects in the presence of estrogen deficiency. As a selective estrogen receptor modulator, phytoestrogens have fewer side effects than estrogens, but their biological activity is influenced by the metabolic pathways *in vivo*, endogenous estrogen levels, and interactions with other drugs and food components. Therefore, the role of phytoestrogens in the prevention and treatment of osteoporosis also needs to be explored.

Single herbal medicines can be developed as preventive or therapeutic agents for osteoporosis due to their multiple anti-osteoporotic active components, but their effects on osteoporosis when used alone as a single botanical agent or in combination with conventional drug therapy still need to be systematically evaluated in the clinical setting. Anti-osteoporotic effects of natural herbal medicines are shown in Table 3.

**TABLE 3:** Anti-osteoporotic effects of natural herbal medicines.

Drugs name	Source	Active ingredients	Mechanism of action	Signaling pathways	Efficacy (Based on *in vitro* and *in vivo* experiments, animal models or cellular models)	Bibliography
Epimedium	Dried leaves of Epimedium, family Berberidaceae	Total Flavonoids Epimedium icariin Epimedium polysaccharide	Promote bone marrow mesenchymal stem cells to osteogenic differentiation, inhibit osteoclast differentiation, and increase bone density	Wnt/β-catenin, MAPK, OPG/RANKL/RANK, BMP/RunX2/OSX, Notch signaling pathway	Nourishing the liver and kidneys, strengthening the muscles and bones, invigorating the blood	Zhao et al. (2019)
*Bone tonic* (*Davallia trichomanoides* Blume)	Dried rhizome of Quercus serrata, family Aquilariaceae	Total Flavonoids Naringenin New North American sage glucoside	Promote osteoblast proliferation and differentiation, regulate cytokines in bone metabolism, and reduce osteoclast activity	OPG/RANKL/RANK, histone K, Wnt/β-catenin, and Bone forming protein pathway	Tonifying the kidneys and strengthening the bones, invigorating the blood and dispelling bone wind	Lei et al. (2021)
Malaytea scurfpea fruit (*Psoraleae Fructus*)	Dried mature fruit of Psoralea vulgaris (Leguminosae)	Isoflavones Psoralen Isosplenetin	Promote osteoblast differentiation and inhibit osteoclast activity Phytoestrogen-like effects	Upward revision of OPG/RANKL ratio inhibition of NF-κB signaling pathway, the PI3k/AKT signaling pathway, activation of MAPK signaling pathway	Tonifies the kidneys and promotes bone healing	Wang et al. (2024)
Eucommia	Bark of Eucommia, family Eucommiaceae	Total Flavonoids Eucommia lignan	Regulation of bone metabolism Increased serum ALP levels, increased femur mass, and Phytoestrogen-like effects	Wnt/β-catenin, BMP/Smad MAPK, OPG/RANKL/RANK, estrogen signaling pathway	Benefiting Qi, strengthening the spleen, tonifying the muscles and bones, invigorating the blood	Feng et al. (2022)
Radix rehmanniae	Rehmannia concoctions	Sitosterol Stigmasterol	Increase osteoblast proliferation and differentiation, promote bone repair, regulate apoptosis, estrogen response process	Targeting IGF1, VEGFA core protein, PI3K-Akt, MAPK signaling pathway	Benefiting the essence and filling the marrow, nourishing Yin and replenishing blood	Xu et al. (2020b)
Radix achyranthis bidentatae	The root of the amaranth plant hyssop	Alkaloid Hyssop polysaccharides,ß-ecdyssterone	Protects bone minerals and increases osteoblast activity	PI3K/Akt JAK/STAT. NF-κB signaling pathway	Tonifying the liver and kidney, strengthening the waist and knees, invigorating blood circulation and removing blood stasis	Lee et al. (2022)
Lycium barbarum	Fruit of Lycium barbarum, family Solanaceae	Isoflavones Lycium barbarum polysaccharide	Enhancement of osteogenic activity and mineralization capacity of bone marrow mesenchymal stem cells	IL-17, TNF, AGE-RAGE, MAPK signaling pathway	Nourishes the liver and kidneys, strengthens the muscles and bones	Luo et al. (2023)
Astragalus	Roots of Astragalus mongolica or Astragalus membranaceus, family Leguminosae	Astragalus polysaccharide	Promotion of osteoblast proliferation and differentiation, inhibition of osteoclast resorption *via* estrogenic pathway, phytoestrogen-like effects	Osteoclast differentiation pathway, IL-17 factor pathway, AGE-RAGE signaling pathway, estrogen pathway BMP-2/Smads signaling pathway	Tonifying Qi and Blood	Kong et al. (2018)
Angelica sinensis	Dried root of Angelica sinensis, family Umbelliferae	Angelica polysaccharide	Improve bone density and quality, increase the number of osteoblasts	OPG/RANKL/RANK pathway, ERK pathway	Tonifying Qi and Blood	Lim and Kim (2014)
Salvia miltiorrhiza	The root of Salvia miltiorrhiza, family Labiatae	Salvia miltiorrhiza polysaccharides Tanshinone Salvia polyphenols	Promote differentiation of MSCs into osteoblasts, promote proliferation and mineralization of osteoblasts, and inhibit apoptosis of osteoblasts	BMP/Smads, Wnt/β-catenin, ERK/, PI3K/AKT signaling pathway, OPG/RANKL/RANK pathway	Promoting blood circulation and eliminating blood stasis, cooling the blood and eliminating carbuncles	Jiang et al. (2019)
*Dipsaci Radix*	Dried roots of the herbal plant himalayan teasel	Total Saponin	Promote differentiation of bone marrow mesenchymal stem cells towards osteoblasts, promote osteoblast differentiation and mineralization, and increase bone density	Promotes BMP-2 protein expression, OPG/RANKL/RANK pathway	Tonifying the liver and kidney, strengthening the muscles and bones, and renewing fractures	Zhang et al. (2019)
Antler	Young unossified densely antlered antlers of stags	Total Peptides Steroids Polyamines	Inhibits osteoclast differentiation and increases bone density	Wnt/β-catenin signaling pathway	Tonifies the vital energy and blood, benefits the essence and strengthens the muscles and bones	Liu et al. (2023)
Cornus officinalis	The dried, ripe pulp of the dogwood plant	Cornus neoside Total glycosides	Promote osteoblast proliferation and differentiation	Regulation of nuclear factor NF-κB TRPV6, TRPV5 channel protein expression	Tonifying the liver and kidney, astringing sperm and fixing deficiency	Tang et al. (2023)

### 4.2 Compound preparations of Chinese medicine

Expert Consensus on Prevention and Treatment of Primary Osteoporosis by Chinese Medicine (2020) primary osteoporosis is classified into six types of evidence, including deficiency of kidney yang, deficiency of liver and kidney yin, deficiency of spleen and kidney yang, deficiency of kidney and blood stasis, weakness of spleen and stomach, and blood stasis and qi stagnation. Chinese medicine treatment of osteoporosis has the principle of combining disease and prevention, and the long-term nature of treatment should be considered according to the patient’s symptoms. Based on evidence-based treatment, multiple Chinese herbs should be combined in appropriate dosage and usage to form a prescription according to the principle of the combination of ruler and minister. For example, among the three clusters of the formulae, the Suberect spatholobus stem, Bone tonic, Dipsaci Radix, and Achyranthes bidentata Blumetonic tonifying the liver and kidney, and strengthen the tendons and bones. Salvia miltiorrhiza, epimedium and safflower invigorate the blood and tonic the blood, dissipate blood stasis and relieve pain (Zhang N. D. et al., 2016).

The combination of these drugs is a combination of kidney tonics and blood invigorating drugs, which can be used as the basic formula for primary osteoporosis. Because of its many components, Chinese medicine compound preparations can prevent and treat osteoporosis with multiple targets and pathways. Clinical practice and animal experiments indicate that TCM formulas provide a definite therapeutic effect on osteoporosis. The active constituents in TCM formulas are diverse in chemical structure, and include flavonoids, lignans, saponins and iridoid glycosides. Antiosteoporotic mechanism of TCM formulas and herbs involves multi regulatory pathways, such as Wnt/β-catenin, MAPK pathway and RANKL/OPG system. The active ingredients in TCM formulas can be developed in combination as potent drugs, which may exhibit better antiosteoporotic effects compared to the individual compound (Zhang N. D. et al., 2016; Gu et al., 2023). Primary osteoporosis evidence type is shown in Table 4.

**TABLE 4:** Primary osteoporosis evidence type (Zhang N. D. et al., 2016; Zhou et al., 2023).

Primary osteoporosis evidence type	Compounding	Efficacy (Based on traditional beliefs)
Kidney Yang Deficiency Evidence	Right Return Pill (Radix rehmanniae, Cornus officinalis, Lycium barbarum, Angelica sinensis, etc.)	Tonifies the kidneys and strengthens the yang, strengthens the muscles and bones
Liver and Kidney Yin Deficiency Evidence	Six-flavored Dihuang Tang (Radix rehmanniae, Cornus officinalis, Common yam rhizome, etc.)	Nourishing the liver and kidney, filling the essence and strengthening the bones
Spleen and Kidney Yang Deficiency Evidence	Tonic Chinese Medicine and Qi Soup (Astragalus, Radix Angelicae Sinensis, Radix Achyranthes Bidentatae)	Tonifies the spleen and kidneys, strengthens the muscles and bones
Kidney deficiency and blood stasis	Tonifying the kidney and invigorating the blood in soup (Radix rehmanniae, Eucommiae, Lycium barbarum, etc.)	Tonifying the kidneys and strengthening bones, invigorating blood circulation and removing blood stasis
Evidence of weakness of the spleen and stomach	Si Jun Zi Tang (Ginseng, Atractylodes, Poria, Licorice, etc.)	Benefiting Qi, strengthening the spleen, tonifying the spleen and stomach
Blood stasis and Qi stagnation	Ginseng and Atractylodes macrocephala (White lentil, Atractylodes, Poria, etc.)	Promoting Qi and Blood circulation, resolving blood stasis and relieving pain

### 4.3 Acupuncture treatment

Drug therapy is limited in clinical application due to its adverse effects and economic burden. Therefore, it is essential to seek the effective and low adverse effect complementary alternative therapy.

Acupuncture therapy is a traditional Chinese medicine treatment for osteoporosis that can improve bone metabolism by increasing bone density in patients and exerting regulatory effects on the major signaling pathways of bone metabolism and key signaling molecules of the pathways. Studies have found that rehabilitation of osteoporosis patients combined with warm acupuncture therapy yielded significant therapeutic effects, reducing pain levels, regulating bone strength and hormone levels (Xu G. et al., 2020). Pan et al. (2018) found that acupuncture combined with Chinese herbal medicine and acupuncture combined with Western medicine were significantly more effective than Chinese herbal medicine combined with Western medicine. However, there are no clear conclusions about the efficacy and safety of acupuncture in the treatment of osteoporosis. There are few clinical studies on acupuncture treatment and the evaluation criteria are relatively simple. There is no more accurate detection index and detection method to evaluate the treatment results. In addition, acupuncture is prohibited for people with coagulation disorders, skin ulcers or infections, and scarred skin, as well as diabetic and leukemia patients.

Combining acupuncture with drug therapy can improve patient symptoms, enhance clinical outcomes, and help promote functional recovery. With the growing trend of combining traditional medicine with acupuncture treatments, it is important to develop a conceptual model of combining Chinese and Western medicine. However, it is important to note that it is important to create a safe and effective model using a scientific evidence-based approach, and to carry out multicentre, large-sample, high-quality randomized clinical controlled trials to provide evidence for acupuncture treatment. This should be done correctly by trained and regulated professionals, avoiding complete faith in treatments based on folk beliefs.

## 5 Discussion

At present, anti-osteoporosis drugs with dual effects of inhibiting bone resorption or promoting bone formation and both are increasingly abundant. The detection of bone biochemical indicators in patients with clinical osteoporosis shows that the decrease of osteoblast function and quantity and the increase of osteoclast function and quantity play different leading roles in the pathogenesis of individual osteoporosis. This provides targeted anti-osteoporosis treatment for patients with osteoporosis of different etiologies. Some new targets for the diagnosis and treatment of osteoporosis, such as serum exosome proteome, have gradually emerged. Humanized IgG2 monoclonal antibody drugs have become a hot spot, providing ideas for the study of new drugs, but hormone replacement therapy and bisphosphonates and other major treatment strategies have more adverse reactions. How to further improve the specificity of targeted drugs to bone tissue and ensure the safety of drugs is the main research direction in the future. With the rapid development of Chinese medicine, the effect of TCM and its compound formulas in the treatment of osteoporosis has gradually become obvious. Single herbs or compound formulations can play an overall regulatory role and can act on osteoporosis in a multi-component, multi-target and multi-systematic manner, and the use of network pharmacology methods can provide assistance in analyzing the active ingredients and pharmacological mechanisms of herbs in disease treatment. However, the evidence for the potential benefits and harms of herbal medicines in the treatment of osteoporosis has not been critically assessed, and there is a lack of high-quality randomized clinical controlled trials. The targeted cells and specific molecular mechanisms for the prevention and treatment of osteoporosis have not yet been fully elucidated, and many studies are still in the primary stage and have not penetrated to the cellular and molecular level, and the principles of herbal medicines in the treatment of osteoporosis still need to be further explored.

Although there are many kinds of new anti-osteoporosis drugs, most of them are still in the experimental stage, and many drugs have not yet established a complete molecular signal network for the treatment of osteoporosis. In addition, the safe and effective dose of drugs suitable for human osteogenesis, the effective route of administration and the effectiveness of local administration also require high-quality experiments for verification and research. With the development of biomaterials and technology, it is necessary to make full use of bone tissue engineering technology in the future, select more suitable models to screen drugs, and make drugs more mature, stable and effective in the field of anti-osteoporosis.
